# Supplementary material for: The role of mainstreamness and interdisciplinarity for the relevance of scientific papers
Source: PLoS One. 2020 Apr 2;15(4):e0230325. doi: 10.1371/journal.pone.0230325 (PMC7117651; doi:10.1371/journal.pone.0230325)
Supplement: S1 Text — (PDF) [file pone.0230325.s001.pdf]

Supplementary Information to: The role of mainstreamness and interdisciplinarity for the relevance of scientific papers

Details of Figure 2

In Fig 2 we visually inspect the situation of how the position of papers is related to their performance shortly (2 years) after publication (b), and within a twenty year timespan (c). Here we provide more detailed information on a few example papers.

The largest node in the lower left cluster (green arrow in Fig 2 (c)) is the Phys. Rev. B article 43.130, entitled "Thermal fluctuations, quenched disorder, phase transitions, and transport in type-II superconductors". Within our scheme, it would classify as a periphery or out-of-the-box paper. It was recognized as important immediately and is still relevant on the long timescale. Many papers in its surrounding, that are more towards the cluster center (many PRLs) got immediate citations but they are not well-cited in the long run. This is seen for example in the Phys. Rev. Lett. 66.953, which appears directly to the southeast of Phys. Rev. B 43.130. Its title is "SQUID picovoltometry of $YBa_2Cu_3O_7$ single crystals: evidence for a finite-temperature phase transition in the high-field vortex state". It was well-cited immediately after publication but lost impact over time; note how the citation rate (node size) reduces from Figs 2b to 2c.

In Fig 2c we mark two review papers with brown arrows. The one that appears in the periphery of the upper right cluster is Rev. Mod. Phys. 63.1 [1]. It explicitly states in its abstract that it uses a variety of methods from different fields, "[...] and rather conventional picture emerges from a number of techniques–analytical (spin-wave theory, Schwinger boson mean-field theory, renormalization-group calculations), semianalytical (variational theory, series expansions), and numerical (quantum Monte Carlo, exact diagonalization, etc.)." This is exactly what is expected. The paper uses methods from various fields and finds a "conventional picture", that is maybe not so far from the mainstream. It is a clear periphery paper.

The other review paper (brown arrow) that appears in the lower right corner of Fig 2c is Rev. Mod. Phys. 63.239 [2]. Its title, "Phenomenological theory of unconventional superconductivity" already hints at its non-mainstreamness. The paper only got significant recognition in the long run. The paper appears as one in a group of several papers that form a small cluster of papers working on similar problems. All of the papers have a large betweenness and distance to their nearest k-means cluster. Most of them did not gain recognition later on, except for Rev. Mod. Phys. 63.239.

Fig A shows the same section of the BC network as in Fig 2b. Node size represents the annual citation rates after two years. In Fig Ab we make all nearest neighbours of a paper (marled by arrow) located in the upper right cluster visible. It is obvious that the marked paper is linked to papers that are located predominantly in the same cluster. Fig Ac shows that the same is true for papers in other clusters; the neighbours of a randomly chosen paper in the lower left cluster are made visible, almost all belong to the same cluster. Finally, in Fig Ad we mark a paper that is situated between the clusters (arrow). Its neighbors in the BC network are clearly papers from both clusters. The marked paper is clearly a bridging paper. Note that not all papers that appear between clusters are bridging papers.

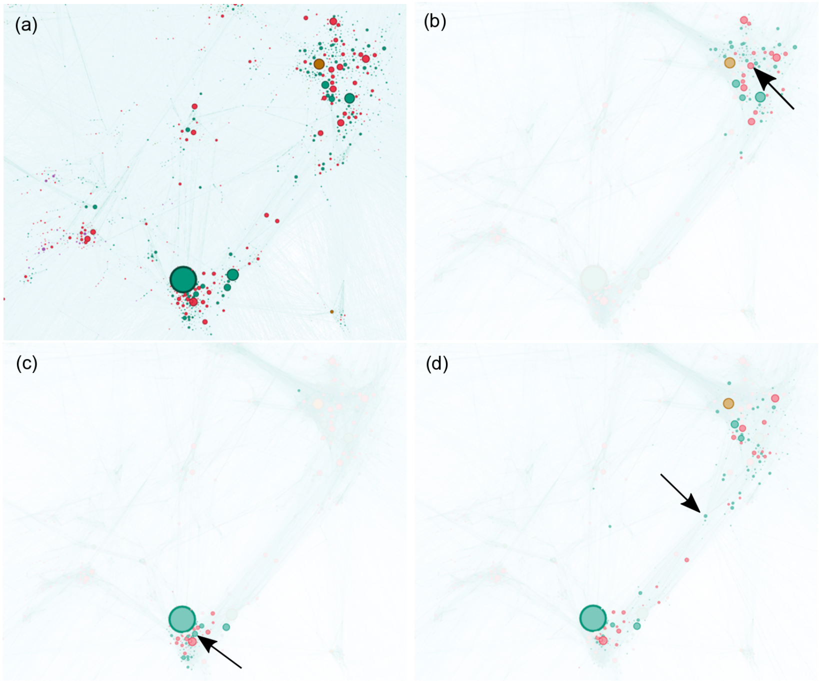

Figure A. (a) Section of the BC network as in Fig 2b. Node size is the short time annual citation rate after two years. (b) If one identifies the nearest neighbours (visible nodes) of a marked paper in the upper right cluster (marked with arrow), it is clear that it is linked to papers predominantly in the same cluster. (c) The same is true for papers in other clusters; neighbours of a paper in the lower left cluster are made visible. They all belong to the same cluster as the marked node. (d) Many papers located between clusters link to papers in the two big clusters. These are bridging papers. Note that not all papers that appear between clusters are bridging papers.

Dependence on other network measures

An obvious candidate measure for interdisciplinarity is the weighted betweenness. In Figs Ba and Bb we show the scatterplot for the twenty year citations, C_i^{20}, versus betweenness, B_i, in the same style as in Figs 3c and 3e. For completeness, we also show the corresponding plots for the closeness, K_i, and the length of reference list, L_i, in Figs Bc and Bd, and Be and Bf, respectively.

Regression analysis

In the regression analysis we use the degree, PACS entropy, I_i, the reference list length, L_i, the closeness centrality, K_i, betweenness, B_i, and distance, D_i, as dependent and the twenty year citation rate, C_i^{20}, as the response variable. Each observation is one paper. First, with a Kolmogorov–Smirnov test we inquire whether to take these variables on a linear or a logarithmic scale (to be closer to a normal distribution). A bivariate linear regression model is then fitted between the response and each dependent variable. Finally, each model receives an additional adjustment term with the reference list length, L_i, to assess the extent to which this variable might confound the observed correlations (length-adjusted model).

Table A shows the results of the regression analysis performed on the 8673 papers in 1991. For results that involve the PACS entropy, I_i, we considered only those 2491

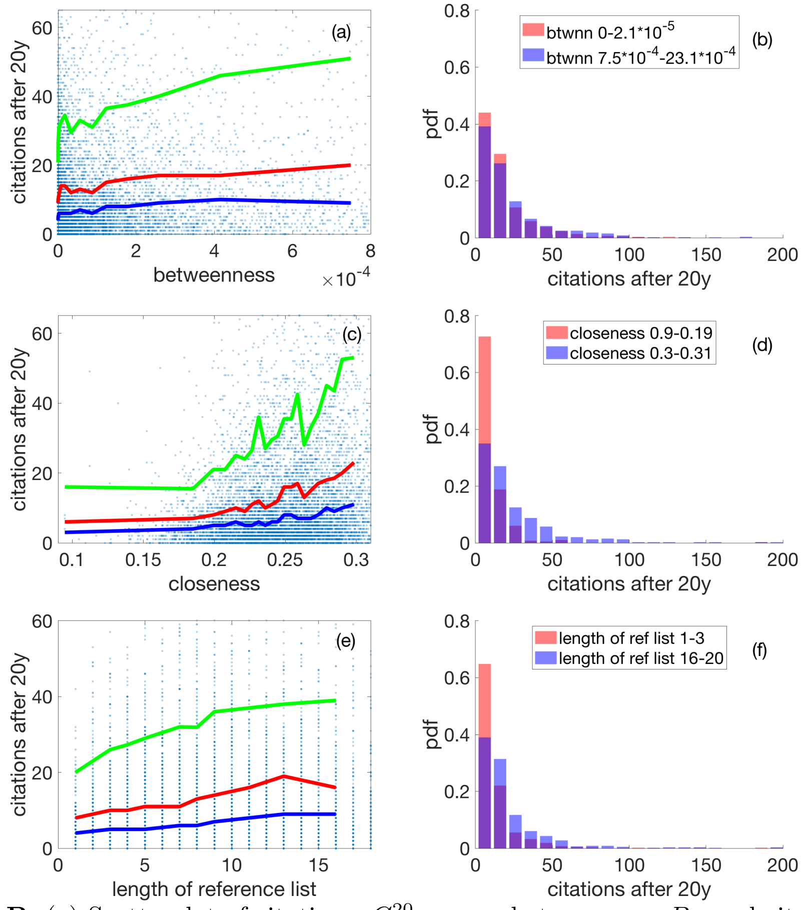

Figure B. (a) Scatterplot of citations, C_i^{20}, versus betweenness, B_i, and citations distributions for small (red) and large values (blue) for betweenness, (c) and (d) show the corresponding results for closeness, K; (e) and (f) show it for the length of reference lists, L_i.

papers for which enough PACS information was available. For each of the dependent variables (degree, entropy, I_i, reference list length, L_i, closeness, K_i, betweenness, B_i, distance, D_i) we report estimates of the coefficients in a linear bivariate regression on the response variable C_i^{20} in the column labeled "bivariate". We then show how these coefficients change after adjusting for the length of the reference list, column "length-adjusted". For completeness, we also show results for regressing the two year, C_i^2, and ten year, C_i^{10}, citation rates on C_i^{20}.

Table A shows the regression results for the author-level analysis. There we consider two adjustment steps, namely (i) the average length of the reference lists of an author's papers, and (ii) the total number of publications.

Author citations

In Fig C we show the dependence of short-term citations of authors. The figure shows the same panels as Fig 5, with the difference that citations of the authors were assessed only 2 years after the time period in which the papers were written (1981-1991).

Fig D shows results for author citations in 2011 for closeness (a), length of reference

Table A. Results of the linear regression analysis for the bivariate and the length-adjusted model. We report estimates together with their standard deviations (SD, numbers in brackets) and p-values against the null hypothesis that the true coefficient value is zero. Variables marked with $*$ were taken on a logarithmic scale.

$C_i^{20*} \sim$	bivariate		length-adjusted ($+L_i$)	
	estimate (SD)	p-value	estimate (SD)	p-value
Degree*	0.33(1)	$< 10^{-217}$	0.31(1)	$< 10^{-104}$
I_i	0.22(2)	$< 10^{-27}$	0.08(3)	0.008
L_i^*	0.24(1)	$< 10^{-115}$		
K_i	0.25(1)	$< 10^{-118}$	0.16(1)	$< 10^{-41}$
B_i	0.10(1)	$< 10^{-21}$	0.04(1)	$< 10^{-4}$
D_i^*	0.26(1)	$< 10^{-132}$	0.18(1)	$< 10^{-46}$
C_i^{10*}	0.969(3)	$< 10^{-256}$	0.969(3)	$< 10^{-256}$
C_i^{2*}	0.807(6)	$< 10^{-256}$	0.795(7)	$< 10^{-256}$

list (c), and the number of papers (e) that individual authors have written in 1981-1991. Panels (b), (d), and (f) show the corresponding distributions for small and large values. Citations show a strong, super-linear increase with closeness (a), while for the length of the reference list and the number of publications the quantiles increase almost linearly.

Table B. Results of the author-level linear regression analysis for the bivariate, length-adjusted, and number-of-paper adjusted model. We report estimates together with their standard deviations (SD, numbers in brackets) and p-values against the null hypothesis that the true coefficient value is zero. Variables marked with $*$ were taken on a logarithmic scale.

citations after after 20y* $\sim$	bivariate		length-adjusted		no.-of-paper-adjusted	
	estimate (SD)	p-value $<$	estimate (SD)	p-value $<$	estimate (SD)	p-value $<$
Degree*	0.313(4)	10^{-256}	0.285(4)	10^{-256}	0.287(4)	10^{-256}
Distance	0.176(4)	10^{-256}	0.0561(9)	10^{-10}	0.164(4)	10^{-256}
Closeness	0.221(4)	10^{-256}	0.177(4)	10^{-256}	0.214(4)	10^{-256}
Betweenness	0.093(4)	10^{-110}	0.025(4)	10^{-7}	0.090(4)	10^{-106}
Length	0.183(4)	10^{-256}			0.172(4)	10^{-256}
No. papers	0.183(4)	10^{-256}	0.173(4)	10^{-256}		

References

1. Manousakis, E. The spin-$\frac{1}{2}$ Heisenberg antiferromagnet on a square lattice and its application to the cuprous oxides. Rev Mod Phys. 1991;63:1.
2. Sigrist, M., Ueda, K. Phenomenological theory of unconventional superconductivity. Rev Mod Phys. 1991;63:239.

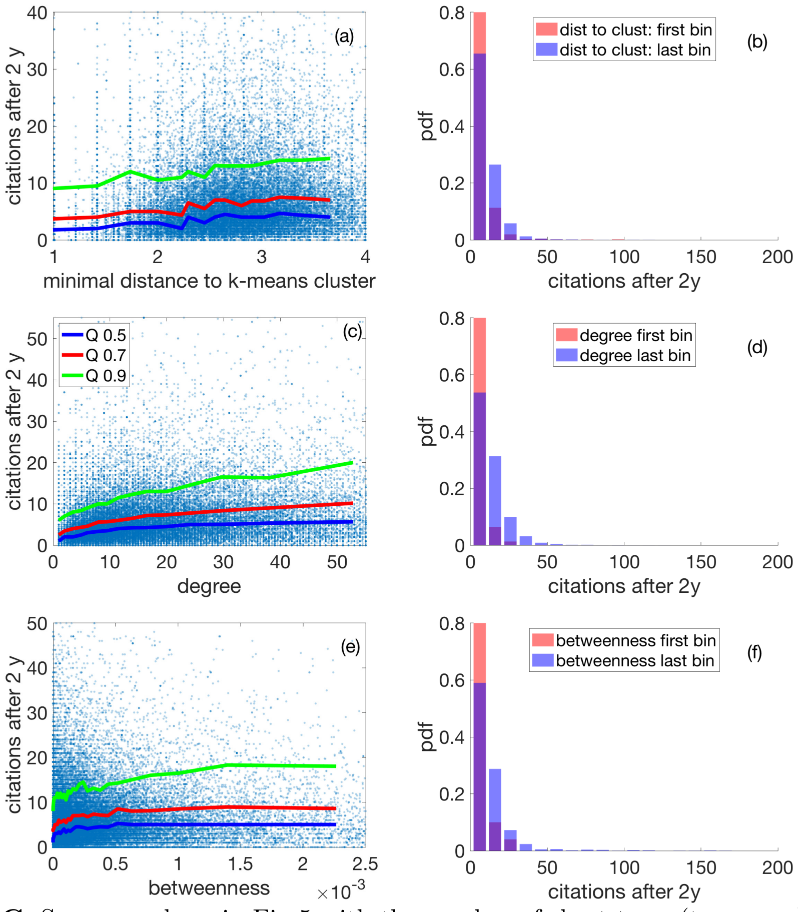

Figure C. Same panels as in Fig 5 with the number of short-term (two years) citations of authors in 1993.

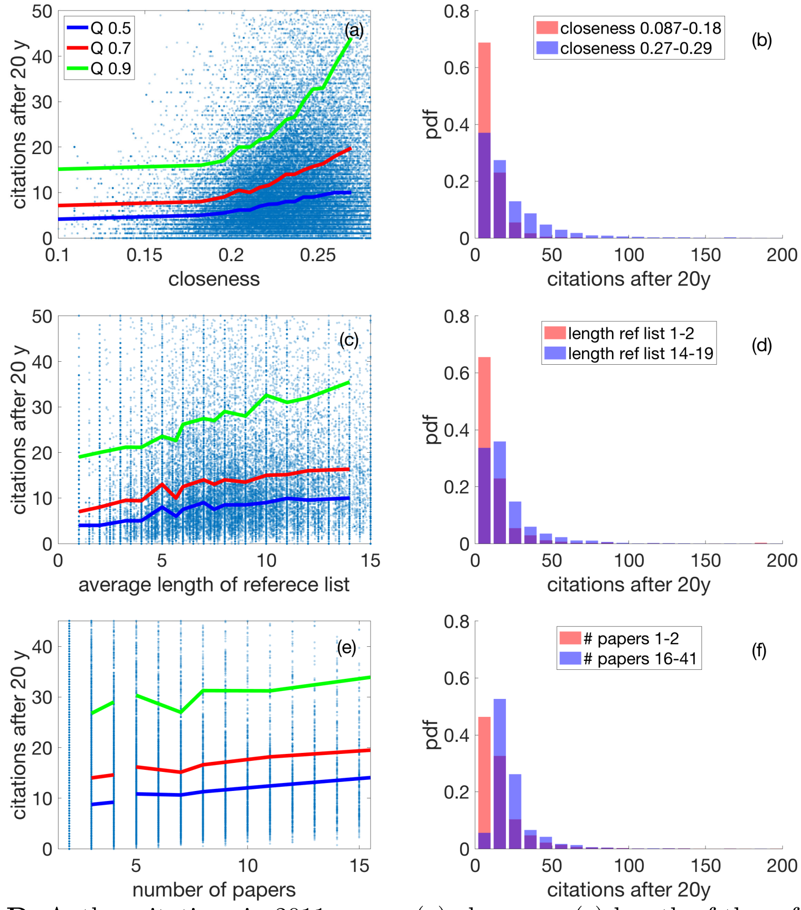

Figure D. Author citations in 2011 versus (a) closeness, (c) length of the reference list, and (e) the number of papers that authors have written in 1981-1991. Every dot represents an author. Panels (b), (d), and (f) show the corresponding distributions for small and large values.
