# Supplementary material for: The role of mainstreamness and interdisciplinarity for the relevance of scientific papers
Source: PLoS One. 2020 Apr 2;15(4):e0230325. doi: 10.1371/journal.pone.0230325 (PMC7117651; doi:10.1371/journal.pone.0230325)
Supplement: Attachment — Submitted filename: responseToReviewers.pdf [file pone.0230325.s002.pdf]

Vienna, Jan 8, 2020

To the
Editorial Board of PLoS one

Subject: Re-submission of manuscript 'The role of mainstreamness and interdisciplinarity for the relevance of scientific papers'

Dear Editors,

thank you for the positive reports. Please find enclosed our revised manuscript 'The role of mainstreamness and interdisciplinarity for the relevance of scientific papers' that fixes the minor problems raised, see the detailed description of changes. We hope that the paper can be accepted in this revised form.

Data are owned by a third-party organization, the American Physics Society. We can not directly share the data with other researchers. However, APS make the data available for research, under data-requests@aps.org, however one must request access on their webpage, https://journals.aps.org/datasets.

Best regards,
Stefan Thurner

List of detailed changes

Referee 1
We thank the referee for the careful reading. We corrected the following issues:

-Figs.3(a) and (b) are not easily readable (in particular Figure 3(a)), since different green scales tend to confuse the reader; maybe it could be better to use just colored line profiles instead of filled boxes.

We made new figures as suggested with line plots.

-References to the SI figures cited in the main text are missed (maybe it is a problem of latex compilation).

Fixed.

-In Ref.[7] the year is missed.

The year is added.

Referee 2
We thank the referee for the careful reading and the nice suggestions. We improved and fixed the following issues:

A remark: I think that interdisciplinary articles can be mainstream as well as out-of-the-box. Interdisciplinarity is defined through the use of several PACS code, through weighted betweenness and also as being intermediate between clusters. Do these definitions coincide?

We agree that interdisciplinary articles can also be mainstream and out-of-the-box. What we are interested here is the focus on the status of new and emerging papers. When we talk about interdisciplinary papers we have in mind young papers, that are found in small clusters between well-established large clusters of mainstream work. In this paper we define interdisciplinarity in three ways, the PACS numbers, the betweenness and the position between clusters. These definitions overlap to varying degrees. While the PACS numbers are self-assigned by the authors, the other two definitions are more "objective" by using the BC network. The idea to include the PACS numbers was mainly to obtain an independent check for the validity of the results. The other definitions are strongly related. Typically, papers with high betweenness will also show a position between clusters. We now mention these issues in the text.

How is the center of a cluster defined? And how the periphery?

The center of a cluster is an output of the k-means algorithm. The periphery is just defined as a large distance to the cluster center. We mention this in the text now.

Page 2 line 54. It is not true that in general 67% of results in science cannot be reproduced. Please qualify this statement (and add reference).

Thank you for pointing out the missing reference. This number was from a 2005 Cochrane study, that was available online, but no-longer is. We fixed the situation, by adding a classical reference and a more recent one, and slightly adapting the text.

Page 3 line 58. Write h-index (not h-factor)

Fixed.

Page 3 The authors discuss reasons why most scientists prefer incremental research. Yet, they do not mention that only few have the intellectual capacity to tackle the hard problems (most of us are not Einsteins).

We fully agree with the referee. However, we would like to not make that comment in that clarity, as it could be seen as being offensive by some. We already have put in the same meaning softer by saying: —*most likely because a methodological framework or the technological means are not yet there*— "Technological means" could have the meaning of missing "technical skills", or non-Einsteinness.
Page 6 line 143 typo: interdiciplinary

Fixed.

Some references to figs in the SI are missing (??)

Problem fixed, see also Referee 1.

Page 9 line 275 Science of science is not a new field (although there is a revival of this term nowadays). Price already used this term and the term remained popular in China over the years.

Thank you for making us aware, and excuse our ignorance. This is now mentioned correctly and the reference is in the text.

Page 10 line 297 typo "fi" must be "for"

Fixed.
